# Facilitation Drives the Positive Effects of Plant Richness on Trace Metal Removal in a Biodiversity Experiment

**DOI:** 10.1371/journal.pone.0093733

**Published:** 2014-04-02

**Authors:** Jiang Wang, Yuan Ge, Tong Chen, Yi Bai, Bao Ying Qian, Chong Bang Zhang

**Affiliations:** 1 School of Life Science, Taizhou University, Linhai, China; 2 Bren School of Environmental Science and Management/Earth Research Institute, University of California Santa Barbara, Santa Barbara, California, United States of America; Key Laboratory of Tropical Forest Ecology, Xishuangbanna Tropical Botanical Garden, Chinese Academy of Sciences, China

## Abstract

**Background:**

Phytoextraction is an environmentally acceptable and inexpensive technique for mine tailing rehabilitation that uses metallophyte plants. These plants reduce the soil trace metal contents to environmentally acceptable levels by accumulating trace metals. Recently, whether more trace metals can be removed by species-rich communities of these plants received great attention, as species richness has been reported having positive effects on ecosystem functions. However, how the species richness affects trace metals removal of plant communities of mine tailing is rarely known.

**Methodology/Principal Findings:**

We examined the effects of species richness on soil trace metal removal in both natural and experimental plant communities. The root lengths and stem heights of each plant species were measured in order to calculate the functional diversity indices. Our results showed that trace metal (Cu, Cd, Pb and Zn) concentrations in mine tailing soil declined as species richness increased in both the natural and experimental plant communities. Species richness, rather than functional diversity, positively affected the mineralomass of the experimental plant communities. The intensity of plant-plant facilitation increased with the species richness of experimental communities. Due to the incremental role of plant-plant facilitation, most of the species had higher biomasses, higher trace metal concentrations in their plant tissues and lower malondialdehyde concentrations in their leaves. Consequently, the positive effects of species richness on mineralomass were mostly attributable to facilitation among plants.

**Conclusions/Significance:**

Our results provide clear evidence that, due to plant-plant facilitation, species richness positively affects the removal of trace metals from mine tailing soil through phytoextraction and provides further information on diversity conservation and environmental remediation in a mine tailing environment.

## Introduction

Research into understanding how species diversity affects ecosystem function has increased over the past two decades [1], [2]. Many studies have shown that species richness has positive effects on ecosystem functions and services, particularly in stressful environments [3]–[9]. Mine tailings are stressful environments characterized by high concentrations of trace metals and low pH, fertility and water retention capacity [10]. Phytoextraction is an environmentally friendly and inexpensive technique for mine tailing rehabilitation that uses metallophyte plants. They accumulate trace metals through their roots, which reduces trace metal content in the soil to environmentally accepted levels [11]. Li et al [12] found that increased algal species richness led to a rise in cadmium removal in an algal microcosm experiment. However, surprisingly few studies have investigated whether species-rich plant communities remove soil trace metals more efficiently than species-poor communities on mine tailing sites.

Previous studies have suggested that the positive effects of species richness on ecosystem functions generally arise through three primary mechanisms: selection effect, niche complementarity and facilitation [13]. Selection effect refers to the increased probability that a randomly assembled species-rich community will contain a species or combinations of species that have the higher than average biomass trait [14]. Niche complementarity and facilitation affect the collective performance of a community through either resource partitioning or positive interactions between species [15]. The two latter mechanisms are called the niche complementarity effect [16]. The selection and complementarity effects are not exclusive and operate simultaneously to bring about changes in ecosystem functions [17]. The principal mechanism behind the positive effects of biodiversity on ecosystem function has been actively debated [18]–[20].

Severely polluted environments, such as mine tailing sites, put ecosystems under constant stress [21]. The central role of facilitation among higher plants in maintaining ecosystem functions in stressful environments has been widely recognized [22]–[25]. However, the importance of this facilitative role may depend, to a large degree, on the characteristics of the species being tested, the stress intensity and the ecosystem function measure being considered [23], [26]–[27]. Moreover, recent studies suggest that the role of facilitation may actually decrease in exceptionally severe environments [28]–[29]. So far, the role played by facilitation in plant communities growing on mine tailings and the underlying mechanism driving it remains unknown.

In a recent study, we found that more diverse natural plant communities led to lower trace metal (Cu, Cd, Pb and Zn) concentrations in mine tailing soil [30]. The results suggested that species-rich plant communities would be more efficient at removing toxic trace metals from mine tailing soils than those with fewer species. Although natural plant communities are more realistic, they hardly allow identifing why the species-rich plant communities having high efficiency as natural plant communities have non-uniform initial conditions, such as different seed pools, soil trace metal concentrations and other soil characteristics. Compared to the natural plant communities, experimental plant communities are more suitable for exploring the underlying mechanism. In this study, we combined both approaches. We aimed to determine: (1) whether plant species richness has a positive effect on the removal of trace metals in mine tailing soil; (2) if a positive effect exists, whether it is mainly attributable to facilitation among plants and (3) what is the mechanism driving the facilitation effects.

## Materials and Methods

### Ethics Statement

No specific permissions were required for this research. The natural plant community experiment was conducted on Huangyan Pb/Zn mine tailings. The exploitation and processing of minerals from the Huangyan Pb/Zn mine stopped about 20 years ago and now plants spontaneously grow on the mine tailings. Consequently, this site is open and no permissions were required to conduct the experiment. The experimental plant community experiment was located at Taizhou University. Some of the authors of the manuscript are Taizhou University staff and the site used for the study was at the authors' experimental research center. Consequently, no specific permissions were needed to conduct the experimental trial on this site. The plant species used in this study were not endangered or protected species.

### Natural Plant Communities

A plant species survey was conducted at the Huangyan Pb/Zn mine tailing site (28°34′23″N, 120°53′44″E) on April 14, 2008 (Table S1 in File S1). Based on the natural species composition data, we designed four species richness levels (1, 2, 4 and 8) for the natural plant communities on the Huangyan Pb/Zn mine tailings. For each species richness level, five plots (1 m×1 m) with different species compositions were randomly selected (Table 1). In each plot, the aboveground and belowground plant material was collected and sorted into species. The plant materials were thoroughly washed with running tap water and rinsed with distilled water to remove any soil particles attached to the plant surfaces. The aboveground and belowground plant materials were separated and oven dried (80°C) to a constant weight and then weighed.

**Table 1:** The species composition of each plot at the four different species richness levels for the natural communities growing on the Huangyan Pb/Zn mine tailings.

Species richness			
1	2	4	8
MF	MF+RB	LC+RB+RP+SC	GP+PA+XS+CC+MF+BP+SC+RP
RB	RP+SN	SC+RL+XS+MF	VH+LP+DL+RP+MF+XS+LF+RL
LC	MF+WJ	SC+MF+CP+LC	SC+RL+XS+RP+LF+RC+MF+RB
XS	GP+RB	LC+MF+GP+LF	XS+RP+LJ+AE+LC+MF+RB+CA
DL	SC+GP	MF+RL+GP+XS	SC+LC+WJ+VH+RP+MF+LF+RB

Footnotes: Species abbreviations and their corresponding full names are listed in Table S1.

In each plot, five soil samples were randomly collected using a soil core sampler (64 mm in diameter×100 mm in length). Each soil sample was collected at a depth of 0–20 cm, air-dried at room temperature, homogenized and sieved (<2 mm). They were then used to determine the soil trace metal concentrations (Cu, Cd, Pb and Zn) and total N. After digestion with HCl and HClO_4_ (4∶1, v/v) [31], the soil trace metal concentrations were measured using an Inductively Coupled Plasma Optical Emission Spectrometer (ICP/OES, Optima 2100DV, Perkin Elmer, USA). Total N in the filtrates was determined using the Berthelot reaction method [32]. Briefly, 1.0 g of K_2_SO_4_ catalyst mixture and 5 ml of concentrated H_2_SO_4_ were added to 0.5 g of air-dried ground tailings in 10 ml digestion tubes. Exactly 20 ml of distilled water was then added to the tubes, which were then filtered. After full digestion, the filtrate and the contents of the tubes were transferred to 50 ml volumetric flasks for the Berthelot reaction.

### Experimental Plant Communities

In order to construct the parallel experimental plant communities, eight species (*Bidens pilosa* Linn, *Phytolacca americana* Linn, *Commelina communis* Linn, *Mirabilis jalapa* Linn, *Chenopodium ambrosioides* Linn, *Solanum nigrum* Linn, *Brassica campestris* Linn and *Xanthium sibiricum* Patrin ex Widder) were randomly selected from the species pool of plants found growing on Huangyan Pb/Zn mine tailings (Table S1 in File S1). *P. americana* is a perennial herbaceous plant and the other seven species are annual herbaceous plants. Seeds from the eight species were collected from the plants growing on the Huangyan Pb/Zn mine tailings, then sown in trays between the 5^th^ and 7^th^ April, 2009 and transplanted about two months after germination. Mine tailing soil samples were randomly collected from the Huangyan Pb/Zn mine tailings and then mixed together to make a composite sample (Tailing properties: organic matter, 330±71.2 mg·kg^−1^; total P, 50.3±10.6 mg·kg^−1^; total N, 120.7±41.4 mg·kg^−1^; Cu, 31.29±3.81 mg·kg^−1^, Cd, 8.92±2.32 mg·kg^−1^; Pb, 924.57±61.23 mg·kg^−1^ and Zn, 1312.45±52.44 mg·kg^−1^).

The seedlings from the eight species were transplanted into plastic containers (pots, 80×80×60 cm^3^) filled with mine tailing soil between June 2^nd^ and 4^th^, 2009 and placed outdoors. We constructed 23 different communities and created four species richness levels (Table 2): eight monocultures, five polycultures of two species, five polycultures of four species and five polycultures of eight species, which were replicated five times. Thirty-two plant seedlings were transplanted into each pot. The plant density was similar to that of the natural plant communities growing on Huangyan Pb/Zn mine tailings. The species in the polycultures were planted at equal densities. A random selection of species from the total pool was not used because, with a limited number of communities, equal representation of the species at each species richness level was not guaranteed. Instead, the species compositions were chosen to guarantee that every species would be selected at least once at each of the four species richness levels. The pots were weeded weekly. In addition to natural rainfall, water was added by spraying the pots during dry periods.

**Table 2:** The species composition of each pot at the four species richness levels for the experimental plant communities.

Species richness			
1	2	4	8
BP	BP+XS	BP+CC+CA+SN	BP+PA+CC+MJ+CA+SN+BC+XS
PA	PA+MJ	PA+MJ+CA+BC	BP+PA+CC+MJ+CA+SN+BC+XS
CC	CC+CA	XS+PA+SN+MJ	BP+PA+CC+MJ+CA+SN+BC+XS
MJ	BP+PA	PA+BP+CC+BC	BP+PA+CC+MJ+CA+SN+BC+XS
CA	SN+BC	BP+CC+MJ+CA	BP+PA+CC+MJ+CA+SN+BC+XS
SN			
BC			
XS			

Footnotes: Species abbreviations and their corresponding full names are listed in Table S1.

The experimental plant communities were harvested on May 10^th^, 2010. All the aboveground and belowground biomasses (living plants) were sorted into species, dried and weighed separately. Before biomass harvesting, a water treatment experiment was conducted. The water content of each pot was determined 5 days after it received the same amount of water through artificial spraying (no water was added during the final 5 days before harvesting). Five soil cores, to a depth of 0–20 cm, were randomly collected from each pot and then combined into one sample. Soil water content was calculated by comparing the wet and dry soil masses (mass of wet soil − mass of dry soil)/mass of dry soil). A proportion of each soil sample was air-dried at room temperature, homogenized and sieved (<2 mm) in order to determine the soil trace metal concentrations (Cu, Cd, Pb and Zn) and total N. The soil trace metal concentrations and total N were measured using the same method employed in the natural plant communities experiment.

At each species richness level, five surviving plants were chosen to determine the malondialdehyde (MDA) concentration in the leaves and the trace metal (Cu, Cd, Pb and Zn) concentrations in plant tissues. Generally, one surviving plant of each species was selected in each pot. However, because some species were not present in all the pots, two or three surviving plants were selected from one pot to guarantee five surviving plants per species richness level. Three surviving plants of *X. sibiricum* were selected at species richness levels two and four, as only three plants of *X. sibiricum* survived. *B. campestris* was not involved in the MDA and trace metal analyses because it did not survive in most of the pots. MDA concentration is an indicator of lipid peroxidation, which reflects the damage to plant tissues caused by oxidative stress [33]. The uppermost five leaves of each surviving plant were used to determine the MDA concentration by using the method followed by Heath and Packer [34]. The dried plant tissues were ground and mixed fully in order to determine the trace metal concentrations. After digestion with HNO_3_ [31], the trace metal concentrations in the plant tissues were measured using an Inductively Coupled Plasma Optical Emission Spectrometer (ICP/OES, Optima 2100DV, Perkin Elmer, USA). The biomass used to measure the MDA was included in the plant biomass of each pot during data analysis.

### Statistical Analysis

The total trace metal content accumulated in the plants (mineralomass) was used to reflect the trace metal removal efficiency by the plants. The mineralomass of a species in one pot was calculated as follows:



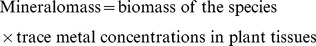



The mineralomass of the plant communities in each pot was the sum of the mineralomasses of all the component species.

Functional diversity indices were represented by the community-weighted mean trait values [35] and the functional diversity Q index [36]. For each species, five plants in monoculture were randomly selected and their root lengths and stem heights were measured with a ruler. The community-weighted mean trait values for each trait were calculated for every pot (*n* = 15) using species trait values and species relative abundances (calculated from the species biomass) following Garnier et al. [35]:
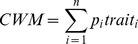
where *n* is the number of species in the pot, *p_i_* is the relative abundance of species *i* and trait*_i_* is the trait value of species *i*.

The average values of the root lengths and stem heights for each species were used to calculate the Euclidean distance between species [37]. *B. campestris* disappeared from all the plots so its root lengths and stem heights were not measured. Following the method used by Heemsbergen et al. [38], the trait values of seven species were put into a number of classes (the highest value was defined as the seventh class and the lowest value was defined as the first class). The Euclidean distance was calculated as follows:
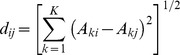
where 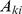 and 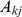 are the class number of species 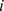 and 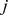 for trait 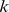 and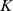 is the number of traits measured (*K* = 2).

The functional diversity Q index was calculated as follows:
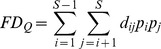
where *S* is the number of species observed in the plot and *p_i_* and *p*_j_ are the relative abundance of species *i* and *j*, respectively.

We used the additive partitioning method to quantify the selection and complementarity effects [16]. The complementarity effect and the selection effect were calculated as follows:
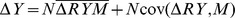
where 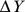 is the net diversity effect, 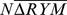 is the complementarity effect and 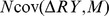 is the selection effect. *N* is the number of species in the polyculture, 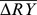
is the average change in relative mineralomass for all species in the mixture and 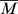 is the average mineralomass of the monocultures. The 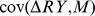 term is the covariance between the mineralomass of the species grown in monocultures (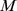) and the change in their relative mineralomass when grown in polycultures.

The relative interaction intensity index (RII) was defined as follows [39]:
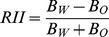
where *Bo* is the mass potentially achieved by the target plant growing in the absence of inter- or intraspecific interactions, i.e. the expected biomass which is the monoculture biomass divided by the species richness of the polycultures. and *Bw* is the mass observed when the target plant was growing with other plants, i.e. the observed biomass of species in the polycultures. This index represents the ratio of the net mass loss/gain due to the interaction relative to the mass affected by only facilitative interaction and competitive interactions. RII has values ranging from −1 to 1, is symmetrical around zero and is negative for competition and positive for facilitation.

The data of soil trace metal concentration in the natural communities were log transformed to meet the assumptions for linear regression. We first used simple linear regression to examine the effect of species richness on soil trace metal concentrations (Cu, Cd, Pb and Zn) in the natural and experimental plant communities, respectively, and to examine the effect of species richness on the RII. Correlation analysis (Spearman coefficient) was used to determine the dependence of the complementarity effect, the selection effect or the MDA concentrations of each species in the experimental plant communities on the actual species richness, and to determine the dependence of biomass on the actual species richness, FD_Q_, CWM_root_ or CWM_stem_. These analyses were performed using the SPSS 11.5 program (SPSS Inc, Chicago, IL, USA) for Windows and significance levels for all analyses were set at *P*<0.05. The multiple stepwise regressions were performed to examine the effects of actual species richness, FD_Q_, CWM_root_ and CWM_stem_ on mineralomass in the polycultures. The optimal model was selected with respect to its value of AIC (Akaike Information Criterion). The model with the lowest value of AIC was selected as the optimal model. For the selected optimal model, the normality of residuals (Shapiro-Wilk test), homosedasticity (Breusch-Pagan test) and multicollinearity among predictors (Variance Inflation Factors) were tested to check for multiple regressions preliminary assumptions. The multiple stepwise regressions were performed using R 2.11.1.

## Results

**Figure 1:**
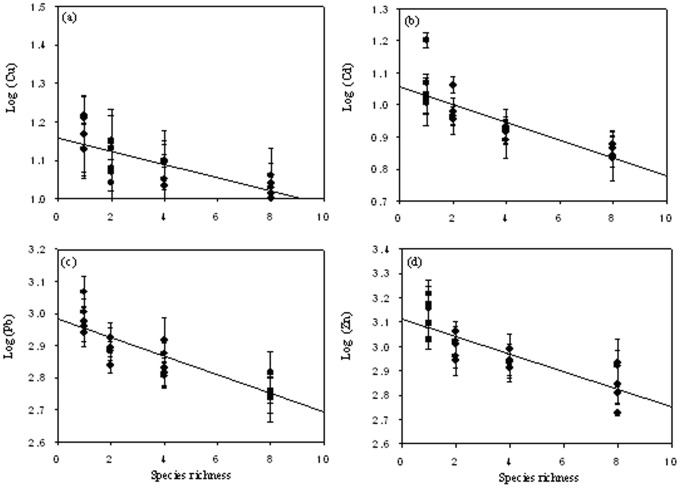
The dependence of soil trace metal concentration on the actual species richness (SR) in the natural plant communities. (a) Fitted line: Log (Cu) = 1.180−0.688 SR, *n* = 25, *P* = 0.001. (b) Fitted line: Log (Cd) = 1.058−0.832 SR, *n* = 25, *P*<0.001. (c) Fitted line: Log (Pb) = 2.981−0.862 SR, *n* = 25, *P*<0.001. (d) Fitted line: Log (Zn) = 3.115−0.815 SR, *n* = 25, *P*<0.001.

**Figure 2:**
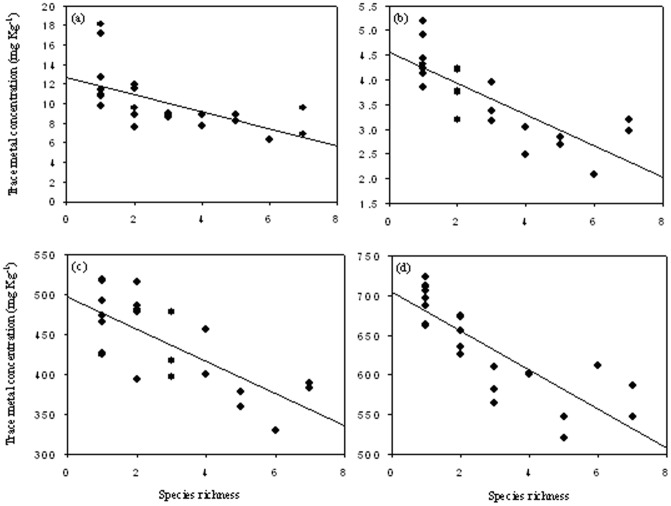
The dependence of soil trace metal concentration (TY) on the actual species richness (SR) in the experimental plant communities. (a) Fitted line: TY_Cu_ = 12.655−0.610 SR, *n* = 23, *P*<0.001. (b) Fitted line: TY_Cd_ = 4.547−0.796 SR, *n* = 23, *P*<0.001. (c) Fitted line: TY_Pb_ = 496.414−0.735 SR, *n* = 23, *P*<0.001. (d) Fitted line TY_Zn_ = 703.839−0.818 SR, *n* = 23, *P*<0.001.

In the natural plant communities growing on the Huangyan Pb/Zn mine tailings, soil trace metal (Cu, Cd, Pb and Zn) concentrations were negatively correlated with species richness (Fig. 1). In the experimental plant communities, even if some plants disappeared in some pots due to the oxidative stress caused by trace metal toxicity, the actual species richness was still significantly and positively correlated with the initial species richness (*r* = 0.966, *n* = 23, *P*<0.001). The trace metal (Cu, Cd, Pb and Zn) concentrations in the soils from the experimental plant communities were negatively correlated with the actual species richness, which was consistent with the results from the natural plant communities (Fig. 2). These results indicated that more diverse plant communities led to lower soil trace metals in the mine tailings.

Actual species richness was always included in the optimal regression model in the multiple stepwise regressions analysis (Table 3), while FD_Q_ was always excluded. CWM_root_ was included for Cu, Cd and Zn, and CWM_stem_ was included for Cd and Pb. Moreover, actual species richness was positively correlated with the biomass of the polycultures (*r* = 0.876, *n* = 15, *P*<0.001). However, FD_Q_ (*r* = 0.430, *n* = 15, *P* = 0.110), CWM_root_ (*r* = 0.494, *n* = 15, *P* = 0.061) and CWM_stem_ (*r* = 0.485, *n* = 15, *P* = 0.067) had no significant relationship with biomass. These results suggested that the significant effect of actual species richness on mineralomass was probably closely linked to biomass production.

**Table 3:** Multiple stepwise regression analyses between mineralomass and explanatory variables from a model that initially contained four variables (actual species richness (SR), FD_Q_, CWM_root_ and CWM_stem_ values for Cu, Cd, Pb and Zn).

Mineralomass	Included variables	VIF	AIC	W	BP
Mineralomass_Cu_	SR	1.13	−55.514	0.919(0.188)	3.706(0.157)
	CWM_root_	1.13			
Mineralomass_Cd_	SR	1.14	−49.798	0.944(0.437)	5.429(0.143)
	CWM_root_	4.86			
	CWM_stem_	4.53			
Mineralomass_Pb_	SR	1.13	−52.161	0.912(0.145)	5.902(0.118)
	CWM_stem_	1.13			
Mineralomass_Zn_	SR	1.13	−52.667	0.903(0.094)	6.032(0.096)
	CWM_root_	1.13			

Footnotes: the values in the brackets are p-value.

**Figure 3:**
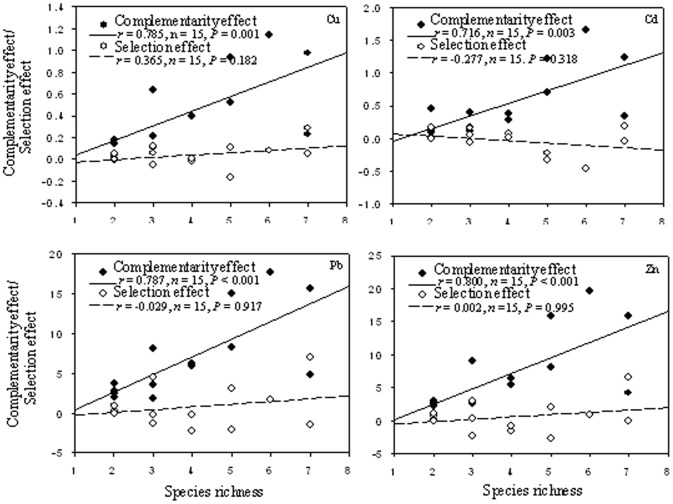
The dependence of the complementarity/selection effect on the actual species richness in the experimental plant communities. a) Cu, b) Cd, c) Pb and d) Zn. The relevant Spearman's correlation coefficient and *P*-value are shown in each panel.

We also investigated whether the complementarity effect or the selection effect led to the positive effects that an increase in actual species richness had on the mineralomasses of Cu, Cd, Pb and Zn. The selection effect had no significant relationship with actual species richness in the polycultures (Fig. 3). The selection effect values varied, ranging from positive to negative across all the experimental plant communities. In contrast, the complementarity effect significantly rose with the increase in actual species richness in the polycultures, compared to the other cultures. Moreover, the complementarity effect values were positive across all the experimental plant communities. Our results suggested that the positive effects of species richness on trace metal accumulations by plants were mainly attributable to the complementarity effect.

**Figure 4:**
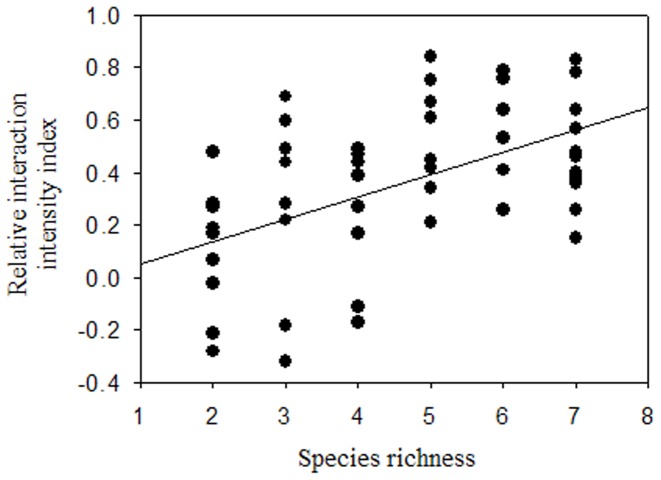
The dependence of relative interaction intensity index (RII) on the actual species richness (SR) in the experimental plant communities. Fitted line: RII = 0.032+0.519 SR, *n* = 57, *P*<0.001.

The relative interaction intensity index (RII) was used to reflect interactions among plants. RII was positive in most polycultures (Fig. 4). Moreover, they significantly increased when actual species richness rose. Moreover, with the exception of *B. campestris*, the increase of survival ratio of other seven plant species increased with the actual species richness (Table 4). The results proved that plant-plant facilitation dominated in the polycultures and increased when the actual species richness rose.

**Table 4:** The survival ratio of each planted species in certain species richness level.

	Planted species richness			
	1	2	4	8
*Bidens pilosa* Linn	0.531	0.563	0.667	0.800
*Phytolacca americana* L.	0.156	0.188	0.250	0.500
*Commelina communis* Linn.	0.438	0.563	0.625	0.900
*Mirabilis jalapa* L.	0.219	0.438	0.500	0.750
*Chenopodium ambrosioides* L.	0.344	0.563	0.500	0.700
*Solanum nigrum* L.	0.156	0.500	0.563	0.650
*Brassica campestris* L.	0.125	0.125	0.063	0.100
*Xanthium sibiricum* Patrin ex Widder	0.250	0.188	0.500	0.550

Survival ratio  =  the survival number of plants in a certain species richness level/the total planted number in the corresponding species richness level.

The malondialdehyde (MDA) concentration directly reflects the oxidative damage to plant tissues caused by trace metals. In the experimental communities, the MDA concentration in the leaves of seven species consistently decreased as actual species richness rose (Table 5). Moreover, the trace metal (Cu, Cd, Pb and Zn) concentrations in the plant tissues consistently increased as actual species richness rose (Figure S1 in File S1). These results suggested that a reduction in oxidative damage to plant tissues probably promoted the accumulation of trace metals.

**Table 5:** The correlations between malondialdehyde (MDA) concentration for each species and the actual species richness in the experimental communities.

Species	*r*	*n*	*P*
BP	−0.900	20	*P*<0.001
PA	−0.660	20	*P* = 0.002
CC	−0.886	20	*P*<0.001
MJ	−0.829	20	*P*<0.001
CA	−0.744	20	*P*<0.001
SN	−0.935	20	*P*<0.001
XS	−0.892	16	*P*<0.001

Footnotes: Species abbreviations and their corresponding full names are listed in Table S1.

## Discussion

Our results demonstrated that species-rich plant communities had lower soil trace metal (Cu, Cd, Pb and Zn) concentrations in both the natural and the experimental plant communities than the species-poor communities. More importantly, we found that species richness had significantly positive effects on mineralomass in the experimental plant communities. Consequently, it is almost certain that the positive effects of species richness on mineralomass led to the lower soil trace metal concentrations seen in the more diverse plant communities.

In theory, the observed positive biodiversity effect could be further partitioned into the selection effect and the complementarity effect [16]. Across all the experimental plant communities, the grand mean value of the selection effect was nearly zero and made little contribution to the positive effect. However, the grand mean value of the complementarity effect was significantly positive and positively correlated with actual species richness. Therefore, the positive effect of actual species richness on mineralomass can mainly be attributed to the complementarity effect. Our results suggest that the positive complementarity effect could be attributed to the increase in biomass (see [40] for more details) and the increases in trace metal concentrations in plant tissues (Figure S1 in File S1) could be due to the increase in actual species richness.

Distinguishing the effects of niche differentiation and facilitation is very difficult in practice [16]. Niche complementarity or facilitation may have important roles in driving the positive complementarity effect seen in the polycultures. Niche differentiation directly linked to interspecific trait differences that are determined by the functional diversity of communities [41]–[42]. In this study, the pot experimental design limited environmental fluctuations and spatial heterogeneity, which are important for resource partitioning between species [17]. Consequently, in this study, functional diversity probably made only a limited contribution to the increased mineralomass. However, environmental fluctuations and spatial heterogeneity are ubiquitous in natural conditions [17]. Consequently, we speculate that niche complementarity may provide more contribution to the positive effect of species richness on mineralomass in natural communities of mine tailings. In the experimental communities, most of species in the polycultures had positive RII values; Most of the polycultures had a larger biomass than their most productive component species in the monocultures (see [38] for more details); Most of plant species (with the exception of *B. campestris*) had higher survival ratio in the polycultures. All of the above results proved plant-plant facilitation dominated in the polycultures, as overyielding [43] and the increase of survival ratio [44] are strong indicators of facilitation. Moreover, the yield and survival ratio increased with the actual species richness. Consequently, the positive complementarity effect seen in the polycultures should be ascribed to the facilitation.

The plants used in the experimental communities are all the established species of the Huangyan Pb/Zn mine tailing. Moreover, their seeds were also collected from the Huangyan Pb/Zn mine tailings. Consequently, the plants used should have high tolerance to the stress of mine tailing. Although mine tailings are characterized by high concentrations of trace metals and low pH, fertility and water retention capacities [10], [30] and mechanically, physically, chemically and biologically restrict plant growth [10], [30], the experimental mine tailing environment may not exert the exceptionally severe stress for these plants. Choler et al. [45] also found, due to lack the experiment at the altitude of >3000 m where should be exceptionally severe for nival communities, the facilitation of the nival communities consistent increased with altitude. Consequently, the role of facilitation decreasing in exceptionally severe environments [28]–[29] was not seen in this experiment.

In this study, the main driving mechanism behind the facilitative processes could be the amelioration of mine tailing soil by the collective performance of all plant species, as soil total N and soil water increased as species richness rose in the experimental communities (see [40] for more details). Plants can improve the soil physical, chemical and biological properties of mine tailings by preventing erosion, adding nutrients and promoting microbial growth and activity [46]. The species-rich experimental communities showed increased biomass production and surface area coverage (data not shown), which likely decreased light penetration and water evaporation and enhance water retention capacity. The root biomass increased as species richness rose (see [40] for more details), which likely increased soil nitrogen due to root exudates. Additionally, soil microbial biomass and activity increased as species richness rose (data not shown), which likely accelerated the decomposition of plant litter and increase the soil nitrogen levels.

Trace metals stress leads to the production of free radicals in plants, which causes lipid peroxidation and tissue damage [47]. MDA is a cytotoxic product of lipid peroxidation [48]. The ameliorated soil properties probably reduced the trace metal stresses, which led to a decrease in MDA concentrations in the plant tissues. Consequently, as actual species richness increased, the plant stress caused by trace metals declined, which may be the reason why trace metal concentrations in plant tissues increased as actual species richness rose.

Taken together, our results provide clear evidence that increased species richness in plant communities exerts a positive effect on soil trace metal removal through increased facilitation among plants present. In comparison to conventional techniques for mine tailing remediation, phytoextraction is cost-effective, safe for humans and environmentally friendly [10], [11]. However, the factors limiting the wider application of this technology involve its relatively low trace metal removal efficiency [49] and the small biomass of most of the hyper-accumulators identified so far [50]–[52]. Our results indicated that diverse plant communities can promote biomass production and trace metal accumulation by plants. However, the selection of species to be used in the construction of plant communities for phytoremediation should be prudent. In this study, *B. campestris* was sensitive to the mine tailing environment and the facilitative effect was not beneficial. Although similar results were found in our natural and experimental plant communities, future studies should be expanded to include realistic plant communities established on different mine tailings in order to determine whether the implications of our results can be extrapolated to realistic phytoremediation programs for mine tailings.

## Supporting Information

File S1 **This includes Table S1 and Figure S1.** Table S1 The name of species identified in the species survey of Huangyan Pb/Zn mine tailing. Figure S1 The heavy metal concentrations in plant tissues of seven species in pots with different actual sepcies richness. a) Cu, b) Cd, c) Pb and d) Zn. Species abbreviations are seen in Table S1. The lines shown in each panel are the regression lines (All the P values of Cu, Cd, Pb and Zn for seven species are lower than 0.05).
(DOC)

## References

[1] Hooper DU, Adair EC, Cardinale BJ, Byrnes JEK, Hungate BA, et al (2012) A global synthesis reveals biodiversity loss as a major driver of ecosystem change. Nature 486: 105–108. 22678289 10.1038/nature11118

[2] Midgley GF (2012) Biodiversity and ecosystem function. Science 335: 174–175. 22246761 10.1126/science.1217245

[3] Kennedy TA, Naeem S, Howe KM, Knops JMH, Tilman D, et al (2002) Biodiversity as a barrier to ecological invasion. Nature 417: 636–638. 12050662 10.1038/nature00776

[4] Cardinale BJ, Srivastava DS, Duffy JE, Wright JP, Downing AL, et al (2006) Effects of biodiversity on the functioning of trophic groups and ecosystems. Nature 443: 989–992. 17066035 10.1038/nature05202

[5] Cadotte MW, Cardinale BJ, Oakley TH (2008) Evolutionary history and the effect of biodiversity on plant productivity. PNAS 105: 17012–17017. 18971334 10.1073/pnas.0805962105 PMC2579369

[6] Kaneryd L, Borrvall C, Berg1 S, Curtsdotter A, Eklöf A, et al (2012) Species-rich ecosystems are vulnerable to cascading extinctions in an increasingly variable world. Ecol Evol 2: 858–872. 22837831 10.1002/ece3.218 PMC3399205

[7] Lei PF, Scherer-Lorenzen M, Bauhus J (2012) The effect of tree species diversity on fine-root production in a young temperate forest. Oecologia 169: 1105–1115. 22298110 10.1007/s00442-012-2259-2

[8] Maestre FT, Quero JL, Gotelli NJ, Escudero A, Ochoa V, et al (2012) Plant species richness and ecosystem multifunctionality in global drylands. Science 335: 214–217. 22246775 10.1126/science.1215442 PMC3558739

[9] Thompson PL, Shurin JB (2012) Regional zooplankton biodiversity provides limited buffering of pond ecosystems against climate change. J Anim Ecol 81: 251–259. 21950456 10.1111/j.1365-2656.2011.01908.x

[10] Meeinkuirt W, Pokethitiyook P, Kruatrachue M, Tanhan P, Chaiyarat R (2012) Phytostabilization of a Pb-contaminated mine tailing by various tree species in pot and field trial experiments. Int J Phytoremediat 14: 925–938. 10.1080/15226514.2011.636403 22908655

[11] Novo LAB, Covelo EF, González L (2013) Phytoremediation of amended copper mine tailings with Brassica juncea,. Int J Min, Reclamat & Environ 27: 215–226.

[12] Li SP, Li JT, Kuang JL, Duan HN, Zeng Y, et al (2012) Effects of species richness on cadmium removal efficiencies of algal microcosms. J Appl Ecol 49: 261–267.

[13] Hector A, Bazeley-White E, Loreau M, Otway S, Schmid B (2002) Overyielding in grassland communities: testing the sampling effect hypothesis with replicated biodiversity experiments. Ecol Lett 5: 502–511.

[14] Cardinale BJ, Gross K, Fritschie K, Flombaum P, Fox JW, et al.. (2013) Biodiversity simultaneously enhances the production and stability of community biomass, but the effects are independent. Ecology. In press. 10.1890/12-1334.1 24015514

[15] Tilman D, Lehman C (2001) Biodiversity, composition, and ecosystem processes: theory and concepts. In: Kinzig AP, Pacala SW, Tilman D, editors. The functional consequences of biodiversity. Princeton: Princeton University Press. pp. 9–41.

[16] Loreau M, Hector A (2001) Partitioning selection and complementarity in biodiversity experiments. Nature 412: 72–76. 11452308 10.1038/35083573

[17] Špaèková I, Lepš J (2001) Procedure for separating the selection effect from other effects in diversity–productivity relationship. Ecol Lett 4: 585–594.

[18] Huston MA, Aarssen LW, Austin MP (2000) No consistent effect of plant diversity on productivity. Science 289: 1255a. 10979839 10.1126/science.289.5483.1255a

[19] Naeem S (2000) Reply to Wardle et al. Bull Ecol Soc Am 81: 241–246.

[20] Fargione J, Tilman D, Dybzinski R, Lambers JHR, Clark C, et al (2007) From selection to complementarity: shifts in the causes of biodiversity–productivity relationships in a long-term biodiversity experiment. Proc R Soc B 274: 871–876. 10.1098/rspb.2006.0351 PMC2093979 17251113

[21] Kondratyev KY, Krapivin VF, Phillips GW (2002) Global Environmental Change: Modeling and Monitoring. Springer, Berlin.

[22] Baumeister D, Callaway RM (2006) Facilitative effects of *Pinus flexilis* during succession: a hierarchy of mechanisms benefits other plant species. Ecology 87: 1816–1830. 16922330 10.1890/0012-9658(2006)87[1816:fbpfds]2.0.co;2

[23] Maestre FT, Bowker1 MA, Escolar C, Puche MD, Soliveres S, et al (2010) Do biotic interactions modulate ecosystem functioning along stress gradients? Insights from semi-arid plant and biological soil crust communities. Phil Trans R Soc B 365: 2057–2070. 20513714 10.1098/rstb.2010.0016 PMC2880128

[24] Odadi WO, Karachi MK, Abdulrazak SA, Young TP (2011) African wild ungulates compete with or facilitate cattle depending on season. Science 333: 1753–1755. 21940896 10.1126/science.1208468

[25] He Q, Bertness MD, Altieri AH (2013) Global shifts towards positive species interactions with increasing environmental stress. Ecol Lett 16: 695–706. 23363430 10.1111/ele.12080

[26] Maestre FT, Valladares F, Reynolds JF (2005) Is the change of plant-plant interactions with abiotic stress predictable? A meta-analysis of field results in arid environments. J Ecol 93: 748–757.

[27] Kawai T, Tokeshi M (2007) Testing the facilitation-competition paradigm under the stress-gradient hypothesis: decoupling multiple stress factors. Proc Royal Soc B 274: 2503–2508. 10.1098/rspb.2007.0871 PMC2274984 17686725

[28] Bruno JF, Stachowicz JJ, Bertness MD (2003) Inclusion of facilitation into ecological theory. Trends Ecol Evol 18: 119–125.

[29] Michalet R, Brooker RW, Cavieres LA, Kikvidze Z, Lortie CJ, et al (2006) Do biotic interactions shape both sides of the humped-back model of species richness in plant communities? Ecol Lett 9: 767–773. 16796565 10.1111/j.1461-0248.2006.00935.x

[30] Wang J, Zhang CB, Ke SS, Qian BY (2011) Different spontaneous plant communities in Sanmen Pb/Zn mine tailing and their effects on mine tailing physico-chemical properties. Environ Earth Sci 62: 779–786.

[31] Mcgrath SP, Cunliffe CH (1985) A simplified method for the extraction of the metals Fe, Zn, Cu, Ni, Cd, Pb Cr, Co and Mn from soils and sewage sludges. J Sci food Agr 36: 794–798.

[32] Page AL, Miller RH, Keeney DR (1982) Methods of Soil Analysis, Chemical and Microbiological Properties. Wisconsin: Madison.

[33] Dhir B, Sharmila P, Saradhi PP (2004) Hydrophytes lack potential to exhibit cadmium stress induced enhancement in lipid peroxidation and accumulation of praline. Aquat Toxicol 66: 141–147. 15036869 10.1016/j.aquatox.2003.08.005

[34] Heath RL, Packer L (1968) Photoperoxidation in isolated chloroplasts 1 kinetics and stoichiometry of fatty acid peroxidation. Arch Biochem Biophys 125: 189–198. 5655425 10.1016/0003-9861(68)90654-1

[35] Garnier E, Cortez J, Billès G, Navas M, Roumet C, et al (2004) Plant functional markers capture ecosystem properties during secondary succession. Ecology 85: 2630–2637.

[36] Rao CR (1982) Diversity and dissimilarity coefficients – a unified approach. Theor Popul Biol 21: 24–43.

[37] Walker B, Kinzig A, Langridge J (1999) Plant attribute diversity, resilience, and ecosystem function: the nature and significance of dominant and minor species. Ecosystems 2: 95–113.

[38] Heemsbergen DA, Berg MP, Loreau M, van HalJR, Faber JH, et al (2004) Biodiversity effects on soil processes explained by interspecific functional dissimilarity. Science 306: 1019–1020. 15528441 10.1126/science.1101865

[39] Armas C, Ordiales R, Pugnaire FI (2004) Measuring plant interactions: anew comparative index. Ecology 85: 2682–2686.

[40] Wang J, Zhang CB, Chen T, Li WH (2013) From selection to complementarity: the shift along the abiotic stress gradient in a controlled biodiversity experiment. Oecologia 171: 227–235. 22791185 10.1007/s00442-012-2400-2

[41] Petchey OL, Gaston KJ (2002) Functional diversity (FD), species richness and community composition. Ecol Lett 5: 402–411.

[42] Petchey OL, Gaston KJ (2006) Functional diversity: back to basics and looking forward. Ecol Lett 9: 741–758. 16706917 10.1111/j.1461-0248.2006.00924.x

[43] Drake JM (2003) Why does grassland productivity increase with species richness? Disentangling species richness and composition with tests for overyielding and superyielding in biodiversity experiments. PNAS 270: 1713–1719. 10.1098/rspb.2003.2423 PMC1691425 12964999

[44] Mulder CPH, Uliassi DD, Doak DF (2001) Physical stress and diversity-productivity relationships: the role of positive interactions. Proc Natl Acad Sci USA 98: 6704–6708. 11371612 10.1073/pnas.111055298 PMC34416

[45] Choler P, Michalet R, Callaway RM (2001) Facilitation and competition on gradients in alpine plant communities. Ecology 82: 3295–3308.

[46] Gómez-Sagasti M, Alkorta I, Becerril JM, Epelde L, Anza M, et al (2012) Microbial Monitoring of the Recovery of Soil Quality During Heavy Metal Phytoremediation. Water Air Soil Pollut (2012) 223: 3249–3262.

[47] Šuran J, Prišć M, Rašić D, Srebočan E, Crnić A P (2013) Malondialdehyde and heavy metal concentrations in tissues of wild boar (*Sus scrofa* L.) from central Croatia. J Environ Sci Heal Part B 48: 147–152. 10.1080/03601234.2013.727672 23305283

[48] Choudhary M, Jetley UK, Khan MA, Zutshi S, Fatma T (2007) Effect of heavy metal stress on proline, malondialdehyde, and superoxide dismutase activity in the cyanobacterium Spirulina platensis-S5. Ecotoxicology and Environmental Safety 66: 204–209. 16600377 10.1016/j.ecoenv.2006.02.002

[49] Cooke SJ, Suski CD (2008) Ecological restoration and physiology: an overdue integration. BioScience 58: 957–968.

[50] Parraga-Aguado I, Gonzalez-Alcaraz MN, Alvarez-Rogel J, Jimenez-Carceles FJ, Conesa HM (2013) The importance of edaphic niches and pioneer plant species succession for the phytomanagement of mine tailings. Environ Pollut 176: 134–143. 23419771 10.1016/j.envpol.2013.01.023

[51] Zhang SJ, Li TX, Huang HG, Zou TJ, Zhang XZ, et al (2012) Cd accumulation and phytostabilization potential of dominant plants surrounding mining tailings. Environ Sci Pollut Res 19: 3879–3888. 10.1007/s11356-012-1060-4 22773333

[52] Sheng XF, He LY, Wang QY, Ye HS, Jiang CY (2008) Effects of inoculation of biosurfactant-producing *Bacillus* sp. J119 on plant growth and cadmium uptake in cadmium-amended soil. J Hazard Mater 155: 17–22. 18082946 10.1016/j.jhazmat.2007.10.107

