# Supplementary material for: Facilitation Drives the Positive Effects of Plant Richness on Trace Metal Removal in a Biodiversity Experiment
Source: PLoS One. 2014 Apr 2;9(4):e93733. doi: 10.1371/journal.pone.0093733 (PMC3973567; doi:10.1371/journal.pone.0093733)
Supplement: File S1 — This includes Table S1 and Figure S1. Table S1 The name of species identified in the species survey of Huangyan Pb/Zn mine tailing. Figure S1 The heavy metal concentrations in plant tissues of seven species in pots with different actual sepcies richness. a) Cu, b) Cd, c) Pb and d) Zn. Species abbreviations are seen in Table S1. The lines shown in each panel are the regression lines (All the P values of Cu, Cd, Pb and Zn for seven species are lower than 0.05). (DOC) [file pone.0093733.s001.doc]

**Supporting information**

**Table S1** The name of species identified in the species survey of Huangyan Pb/Zn mine tailing

**Figure S1 The heavy metal concentrations in plant tissues of seven species in pots with different actual sepcies richness.** a) Cu, b) Cd, c) Pb and d) Zn. Species abbreviations are seen in Table S1. The lines shown in each panel are the regression lines (All the *P* values of Cu, Cd, Pb and Zn for seven species are lower than 0.05).

**Table S1** The name of species identified in the species survey of Huangyan Pb/Zn mine tailing

| The name of species | Abbreviation |
| --- | --- |
| *Lygodium japonicum* (Thunb.)Sw. | LJ |
| *Rubus chingii* HU in journ. | RC |
| *Loropetalum chinensis* (R. Br.) Oliv. | LC |
| *Polygala japonica* Houtt. | PJ |
| *Crataegus pinnatifida* Bge. var. major N. | CP |
| *Rhus chinensis* Mill. var. chinensis. | RCM |
| *Commelina communis* Linn. | CC |
| *Smilax china* L. | SC |
| *Ampelopsis cantoniensis* (HK.et Arn.) Planch. | AC |
| *Parathelypteris glanduligera* (Kze.) Ching. | PG |
| *Lysimachia fortunei* Maxim. | LF |
| *Myrica rubra* Siebold et Zuccarini. | MR |
| *Chenopodium ambrosioides* Linn. | CA |
| *Ardisia japonica* (Hornsted) Blume. | AJ |
| *Vitis hancockii* Hance. | VH |
| *Solanum nigrum* Linn. | SN |
| *Lespedeza pilosa* (Thunb.) Sieb. et Zucc. | LP |
| *Rubus parvifolius* Linn. | RP |
| *Brassica campestris* Linn. | BC |
| *Rosa laevigata* Michx. | RL |
| *Geranium carolinianum* Linn. | GC |
| *Ligustrum quihoui* Carr. | LQ |
| *Premna microphylla* Turcz. | PM |
| *Lonicera japonica* Thunb. | LJT |
| *Selaginella uncinata*(Desv.)Spring. | SU |
| *Bidens pilosa* Linn. | BP |
| *Glochidion puberum* (Linn.) Hutch. | GP |
| *Phytolacca americana* Linn. | PA |
| *Herba patriniae* cum radice. | HP |
| *Rosa bracteata* Wendl. | RB |
| *Viola verecunda* A. Gray. | VV |
| *Mirabilis jalapa* Linn. | MJ |
| *Dryopteris fuscipes* C. Chr. | DF |
| *Woodwardia japonica* (L. F.) Sm. | WJ |
| *Osmunda japonica* Thunb. | OJ |
| *Aster ageratoides* Turcz. | AA |
| *Dicranopteris linearis* (Burm.) Underw. | DL |
| *Sassafras tzumu* (Hemsl.) Hemsl. | ST |
| *Lespedeza chinensis* G. Don. | LC |
| *Adiantum edgewothii* Hook. Sp. | AE |
| *Pinus massoniana* Lamb. | PM |
| *Miscanthus floridulus* (Lab.) Warb. ex Schum. et Laut. | MF |
| *Buddleja lindleyana* Lindley Summerlilic. | BL |
| Mallotus repandus (Willd.) Muell. Arg. | MR |
| *Xanthium sibiricum* Patrin ex Widder. | XS |
| *Vaccinium bracteatum* Thunb. | VB |
| *Agrimonia pilosa* Ledeb. | AP |
| *Mallotus apelta* (Lour.)Muell.Arg. | MA |

**Figure S1** **The trace metal concentrations in plant tissues of seven species in pots with different actual sepcies richness.** a) Cu, b) Cd, c) Pb and d) Zn. Species abbreviations are seen in Table S1. The lines shown in each panel are the regression lines (All the *P* values of Cu, Cd, Pb and Zn for seven species are lower than 0.05).
